# Modeling a multiple-chain emeraldine gas sensor for NH_3_ and NO_2_ detection

**DOI:** 10.3762/bjnano.13.64

**Published:** 2022-07-26

**Authors:** Hana Sustkova, Jan Voves

**Affiliations:** 1 Faculty of Electrical Engineering, Czech Technical University in Prague, Technicka 2, Prague 6, Czech Republic

**Keywords:** ammonia, gas sensor, nitrogen dioxide, numerical computation, polyaniline

## Abstract

This paper describes atomistic device models of a multiple-chain polyaniline (PANI) gas sensing component, utilizing the non-equilibrium Green’s functions formalism. The numerical results are compared with experimental data of ammonia and nitrogen dioxide detection. Multiple molecules of PANI in the form of emeraldine salt were studied with more than one absorbed molecule of ammonia or nitrogen dioxide. From the *I*–*V* characteristics of the system with and without adsorbed gas molecules for gas concentrations of 3, 6, 9, and 12 ppm, the effective resistance changes, (*R* − *R*_0_)/*R*_0_, were obtained and compared with experimental results. A good agreement with the measured values was obtained. In summary, PANI as emeraldine salt was numerically modeled for several adsorbed gas concentrations, several gas configurations, and different PANI molecule positions, including carrier hopping between them. The results are comparable to the experiment and show good properties for the application as gas sensor device for NH_3_ detection and rather good properties for NO_2_ detection.

## Introduction

Polyaniline is a conducting polymer consisting of benzene rings connected by nitrogen units, which can be used in a wide spectrum of applications, for example, dyes for antistatically equipped clothing, capacitors, solar cells, energy storage devices, and polymer light-emitting diodes [1]. One electrical property of PANI are the π-conjugated bonds in the benzene rings. The key to this is the NH group, which can be doped. These nitrogen units are the key element of the chain conductivity of PANI [2]. PANI can be synthesized chemically or electrochemically, with different results in terms of polymer conductivity [3]. There are three different ground states of oxidation, which leads to a large spectrum of the electric properties of PANI.

**Figure 1:**
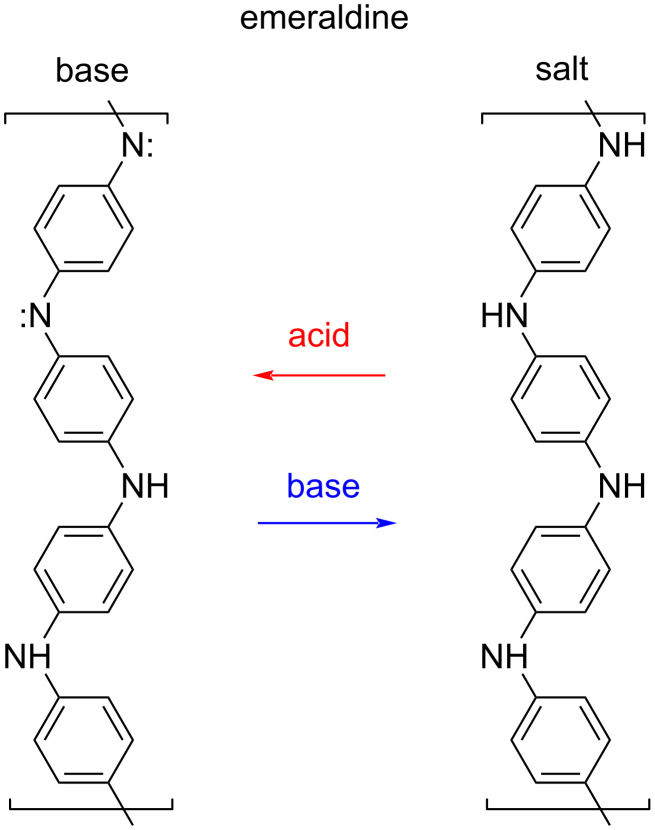
Polyaniline emeraldine base and emeraldine salt.

First, leucoemeraldine, the fully reduced PANI variety, is a transparent polymer and a good electrical insulator, whereas the second one, the base form emeraldine, which is partially oxidized, is blue and insulates worse. The violet PANI variety, fully oxidized and semi-conducting, is named pernigraniline. Considering the insulating or weakly conducting PANI varieties, after inserting a defect into the molecule, one or more polarons can occur. To produce two polarons per one mer consisting of four benzene units, a proton needs to be added to every second nitrogen atom of the emeraldine base, or every second nitrogen atom of the leucoemeraldine base has to be reduced by electrons (Figure 1). Emeraldine base or leucoemeraldine base salts of green color and with conductivity values of up to 400 S/m can be obtained through synthesis [4].

Recently, the polyaniline emeraldine salt has been studied for sensor applications for various gases because it shows a resistance change when gas molecules are in the vicinity of the chain. Interesting for this application could be the detection of NH_3_ or NO_2_ [5].

## Mechanism of Gas Sensing

When gas molecules interact with the PANI chain, the resistance of the bulk material changes. For example, ammonia gas molecules cause an acid–base reaction, a dedoping effect. NH_3_ molecules attract one proton from the doped nitrogen of the PANI chain (NH^+^), resulting in polaron extinction and a new ammonia state, namely 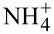. Because these charge carriers are then removed from the PANI chain, the resistance of the polymer increases. The resistance will rise according to the number of nitrogen atoms doped by protons and will be affected by the position and the distance of NH_3_ molecules from the chain. That is, the better the gas can penetrate the polymer structure, the greater the effect will be. The change in resistance is reversible. When the PANI chain without donors is exposed again to air, desorption of the ammonia gas molecules occurs, leading to the return of protons to the nitrogen atoms in the PANI chain. Changing ammonia concentrations cause ammonia molecules to adsorb to or desorb from PANI, yielding a change of resistance [6].

In the work of Kroutil et al. [7], a polyaniline gas sensor setup was used for measuring NH_3_, NO_2_, and other gases. Ammonia should be, according to these results, the gas that most affects the polyaniline resistance; nitrogen dioxide moderately affects the resistance. Many electronic and molecular properties of polyaniline have been studied by quantum mechanical means in [4,8]. The band structure was calculated by Reis et al. [9], together with transmittance, electrical current flow, and charge density. For these calculations, density functional theory (DFT, [10]) based on the generalized gradient approximated (GGA) Perdew–Burke–Ernzerhof (PBE) exchange–correlation functional was used. Guo et al. [5] investigated the graphene/polyaniline adsorption energy of NH_3_, CO, NO, and H_2_ using DFT and molecular dynamics computations. From this, graphene/PANI is highly sensitive to NH_3_ in comparison to the other gases. The density of states, in this case, increases significantly above the Fermi level.

Zhang et al. [11] already modeled sensors detecting single gas molecules using DFT. Also, in our previous work, computations with one emeraldine salt PANI chain and one ammonia molecule were carried out and compared with measurements of 3 ppm ammonia [12]. This paper continues and goes deeper into this issue. It concerns further *I*–*V* characteristic computations of a device composed of several PANI molecules in the presence of several ammonia and nitrogen dioxide gas molecules. The computations used the non-equilibrium Green’s functions formalism (NEGF), and the results were compared with the experiments of Kroutil et al. [7] and Posta and co-workers [13].

## Theoretical Study

Since this article is a continuation to [12], the methods are similar and the computation for this work was carried out using Synopsis QuantumATK [14] as well, in this case, version 2020.09. Taking into account the good accordance of benchmark computed data for polyaniline, semi-empirical methods were used, applying tight bonds. At the beginning of this research in [12], the benchmark was obtained through using the extended Hückel method [15]. It is a fast computation method with very good results for the polyaniline system. This method was used for this paper, too. Computation of the device was performed using NEGF and the extended Hückel method [15] to obtain the transmission of the device system, resulting in the *I*–*V* characteristics.

As already written in [12], calculation of the electron density is based on the occupied eigenstates of the Kohn–Sham Hamiltonian, with the Ansatz of a Fermi–Dirac distribution. QuantumATK divides this density into contributions from “left” and from “right”, while the model is supposed to be in a Cartesian system. The distance between electrodes lies along the *z*-axis and the direction from *z* to −∞ is defined as “left”. Likewise, the direction from *z* to +∞ is defined as “right”. Now, the left density matrix contribution is calculated using NEGF [16]:


[1] $$D^{\text{left}}=\int \rho^{\text{left}}\left(\varepsilon \right)f\left(\frac{\varepsilon -\mu_{L}}{k_{\text{B}}T_{\text{L}}}\right)\text{d}\varepsilon ,$$

and the contribution of the right side is calculated accordingly. In the equation, *f* is the Fermi–Dirac function for the electron distribution in the left or right electrode and depends on the electron temperature *T*_L_; ρ(ε) stands for the spectral density matrix, defined by the broadening function Γ and the retarded Green’s function *G*. The spectral density matrix has the form [16]:


[2] $$\rho^{\text{left}}\left(\varepsilon \right)=\frac{1}{2\pi }G\left(\varepsilon \right)\Gamma^{\text{left}}\left(\varepsilon \right)G^{†}\left(\varepsilon \right),$$

for Green’s function and broadening function


[3] $$G\left(\varepsilon \right)=\frac{1}{\left(\varepsilon +i\delta_{+}\right)S-H}$$

and


[4] $$\Gamma^{\text{left}}=\frac{1}{i}\left[\Sigma^{\text{left}}-{\left(\Sigma^{\text{left}}\right)}^{†}\right].$$

In this equation, δ_+_ is an infinitesimal positive number, Σ is the self-energy of the left or right electrode, *S* stands for the overlap matrix and *H* is the Hamiltonian matrix. These are defined for the whole system [16]. The central region is ruled by the non-equilibrium electron distribution. Using the retarded Green’s and broadening function for the electrodes, the transmission spectrum is modeled as [16]:


[5] $$T\left(\varepsilon \right)=G\left(\varepsilon \right)\Gamma^{\text{left}}\left(\varepsilon \right)G^{†}\left(\varepsilon \right)\Gamma^{\text{right}}\left(\varepsilon \right).$$

## Results and Discussion

**Figure 2:**
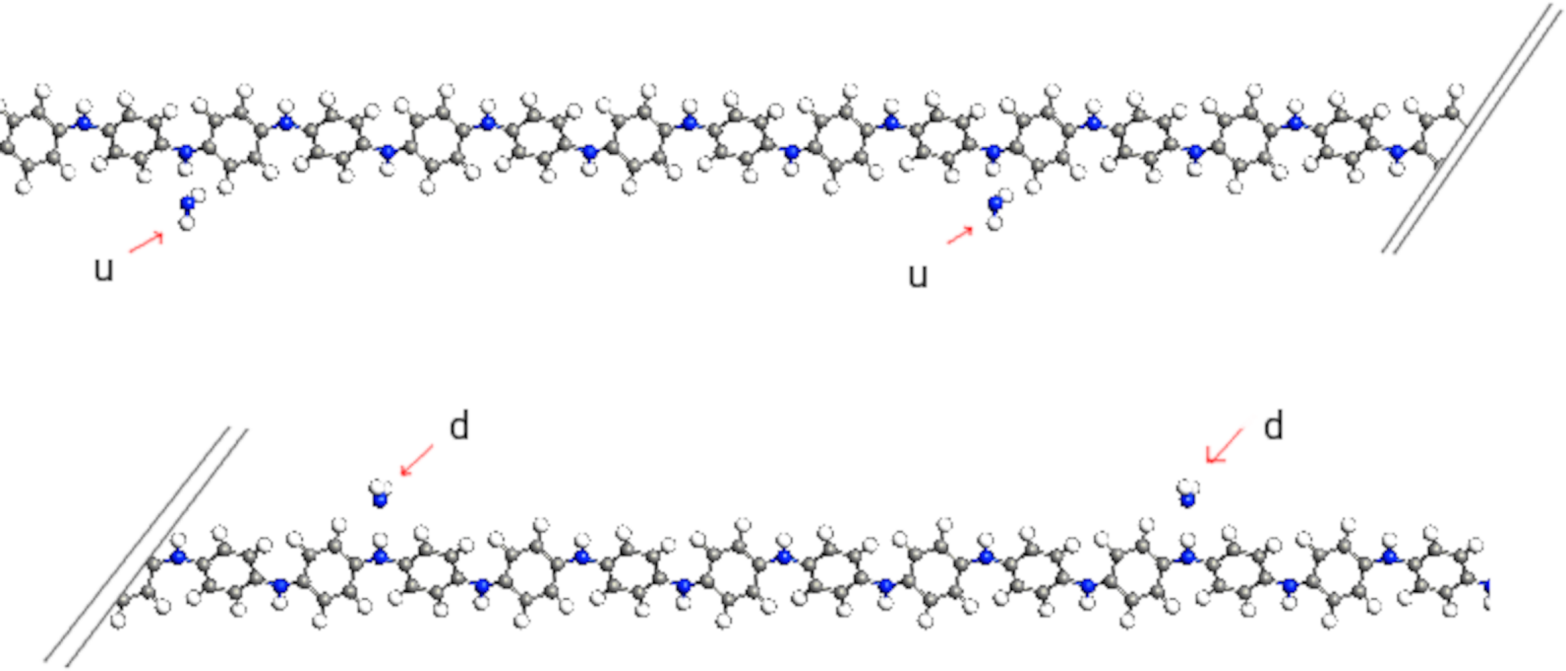
One PANI chain and four NO_2_ molecules. Two in position “d” (doped) and two in position “u” (undoped), also denominated 2D+2U.

**Figure 3:**
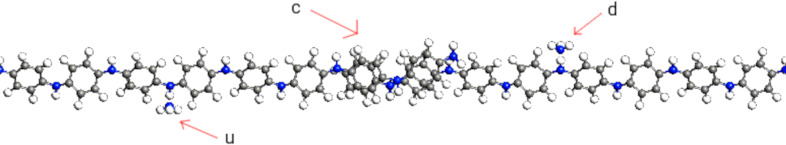
Two PANI chains overlapping in the middle with a gap of 3 Å. The position (c) is for ammonia or nitrogen dioxide molecules. Further positions are doped (d) and undoped (u), shown here with an ammonia molecule.

**Figure 4:**
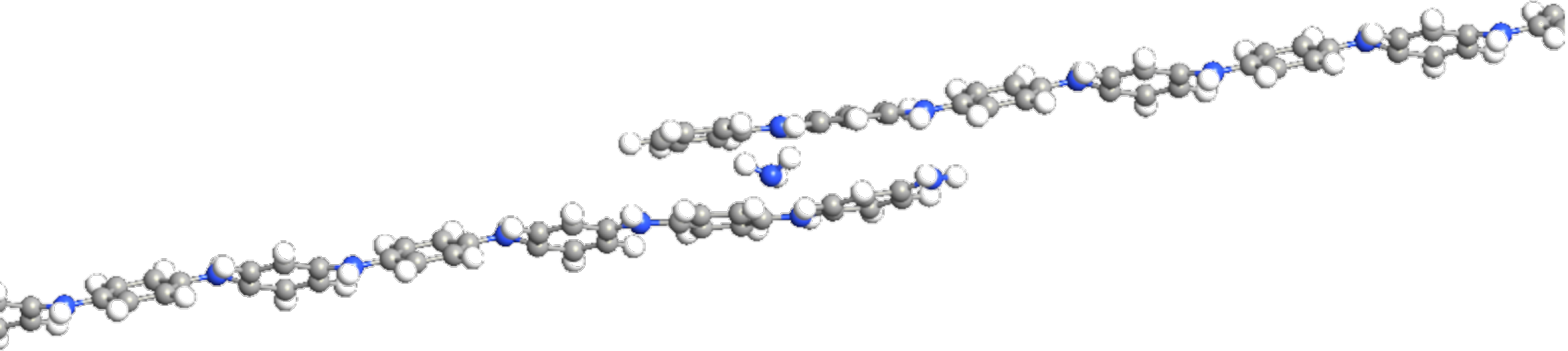
Two PANI chains overlapping in the middle with a gap of 3 Å top view. Between the chains one ammonia molecule is located.

Emeraldine salt molecules were modeled for one chain and for several overlapping chains, both with or without gas molecules. Every second nitrogen in the chain was doped. For the gas molecule interacting with the chain, several positions were considered, namely close to the doped and undoped nitrogen (Figure 2), in the vicinity of the benzene center, and, if several chains were modeled, between two chains (Figure 3 and Figure 4). Also, the orientation of the gas molecule was varied. Apart from these variations, one position and one orientation of the gas molecule was determined utilizing energy minimizing optimization. For all these situations, computation of the transmission spectrum was carried out. Transmission spectra were used to obtain relevant *I*–*V* characteristics.

There were only one or two molecule chains in the calculations and only several ammonia or nitrogen dioxide molecules are affecting the PANI molecules. Therefore, the quantity of free charge carriers is limited and very small in comparison with macroscopic PANI media. This leads to current saturation. The electric resistance was estimated from the initial linear behavior of the *I*–*V* characteristics before saturation.

At first, we have computed only models with one PANI chain. We have chosen fixed positions of ammonia and nitrogen dioxide molecules. We have computed the local energy minima through molecular dynamics, which results in the optimized position and orientation of the gas molecule. The spatial orientation of the gas molecules was estimated by these optimizations. Next, one up to four molecules were placed near the chain according to the optimization, either next to the doped nitrogen atom (D) or next to the undoped one (U). For ammonia, the nitrogen of NH_3_ was 2.09 Å away from the doped/undoped nitrogen of the PANI chain, while the bond length between N and H was 0.9994 Å. For nitrogen dioxide in the position “D”, the distance of N–N was 3.04 Å and the gas molecule angle was 103.904°, while in the position “U”, the N–N distance was 2.65 Å and the gas molecule angle was 103.671°. Resistance values for (i) one gas molecule near the doped nitrogen atom (1D+0U), (ii) one gas molecule near the undoped nitrogen atom (0D+1U), (iii) one gas molecule near the doped nitrogen atom and one gas molecule near the undoped nitrogen atom (1D+1U), and one gas molecule near the doped nitrogen atom and two gas molecules near each undoped nitrogen atom (1D+2U), were estimated from the *I*–*V* characteristics.

**Figure 5:**
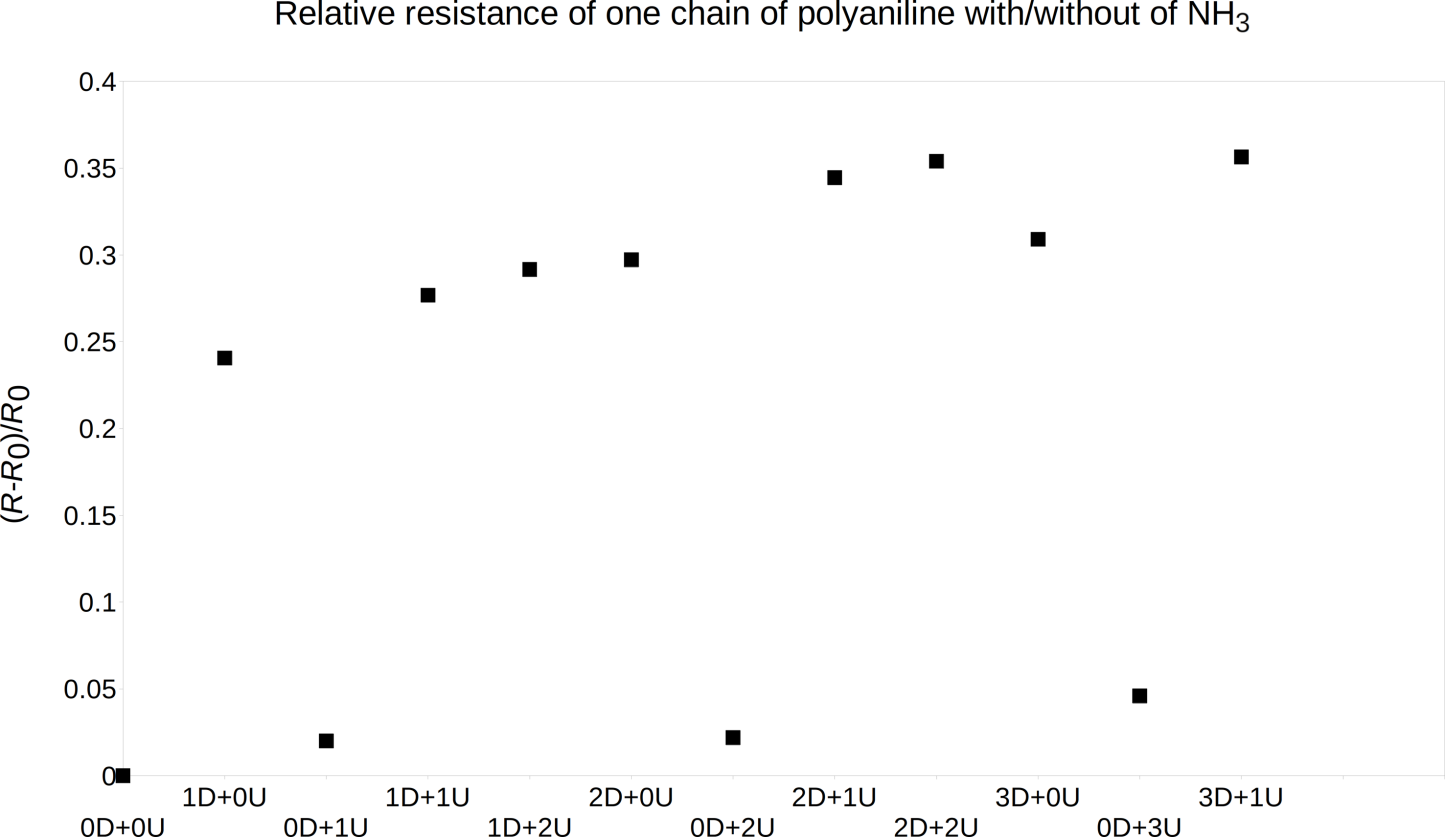
The relative resistance change of emeraldine salt, one molecule chain was taken into account, by the presence of several ammonia gas molecules near the chain, in the positions as described above.

The relative resistance change Res,


[6] $$\text{Res}=\frac{R-R_{0}}{R_{0}},$$

was estimated, where *R*_0_ is the electrical resistance of PANI without any gas molecules and *R* is the electrical resistance of PANI in the presence of ammonia or nitrogen dioxide. The relative resistance change for ammonia gas molecules and one chain of emeraldine salt is shown in Figure 5. One can see, that the main part of the resistance change is due to the ammonia molecule near the doped nitrogen in the polyaniline chain and that the resistance change for more ammonia molecules on different positions (near the doped/undoped polyaniline nitrogen) is like the sum of the resistance changes of both positions. Further, the resistance seems to grow up linearly for the ammonia gas molecule positions.

**Figure 6:**
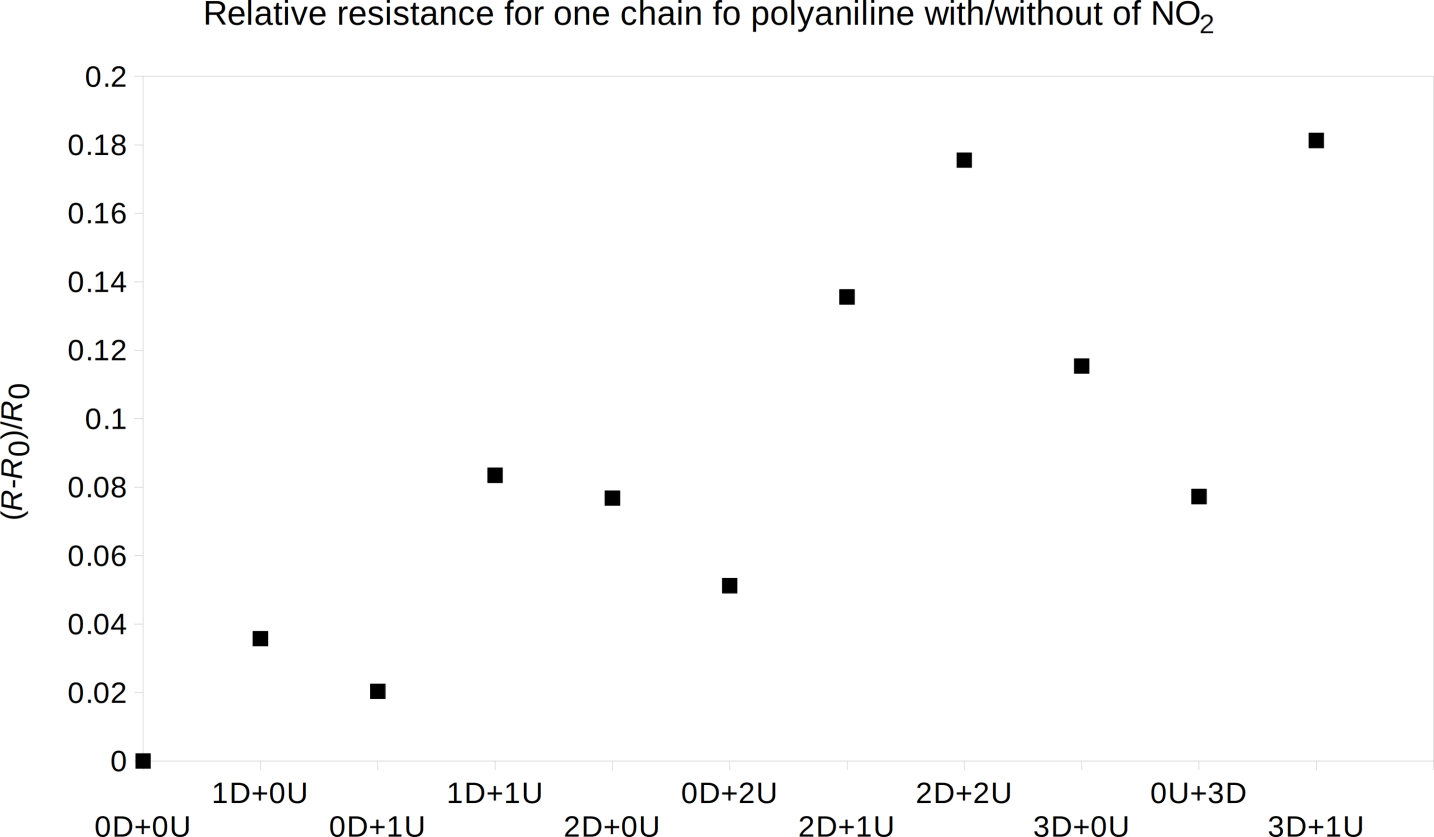
The relative resistance change of emeraldine salt, one molecule chain was taken into account, by the presence of several nitrogen dioxide gas molecules near the chain, as described above.

By the same means, the relative resistance of the polyaniline chain with and without one or more nitrogen dioxide molecules was computed as well. The *I*–*V* characteristics were computed for several molecule positions near the doped and near the undoped nitrogen atoms, see above. The molecule orientation was estimated through molecular dynamics optimization. The resistance from the linear part of the *I*–*V* characteristics was used to estimate the relative resistance, see Equation 6, and was computed for all sets, see Figure 6.

Similar to the case of ammonia, the resistance change depends strongly on the nitrogen dioxide molecule position. A gas molecule near the doped nitrogen makes the polyaniline chain more sensitive than one near the undoped nitrogen. Also, the resistance change of more nitrogen dioxide molecules on different positions (near the doped/undoped polyaniline nitrogen) is rather the sum of the resistance changes of both positions. The increasing linear resistance change for the gas molecule positions is seen as well. These one-polyaniline-chain data were recalculated for different concentrations of 3, 6, 9, and 12 ppm. We have compared them with experimental data, as shown below. The ppm concentration was calculated from the modeled volume with the polyaniline chains and the corresponding amount of gas molecules acting on the chains.

As a next step, sets with two chains of polyaniline were calculated, where (i) no NH_3_ gas molecules, (ii) one NH_3_ gas molecule near a doped nitrogen (1D+0U+0C), (iii) one NH_3_ gas molecule near an undoped nitrogen(0D+1U+0C), (iv) one NH_3_ molecule between the chains in the central benzene region (0D+0U+1C), see Figure 4, and (v) their combinations were modeled. The relative resistance was estimated in the same way as for one chain with ammonia and nitrogen dioxide described above.

**Figure 7:**
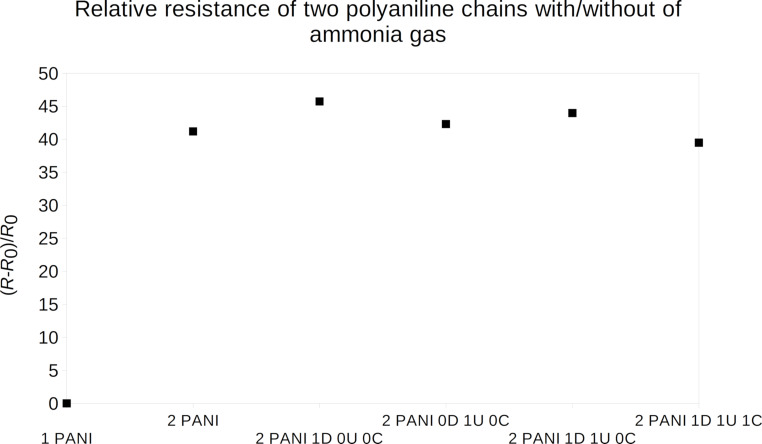
Relative resistance of emeraldine salt in the presence of NH_3_ computed for two overlapping chains of polyaniline, see Figure 4. On the left is the reference value for one chain of polyaniline without any gas molecule.

As a first step, the effect of a discontinued polyaniline chain was studied. The gap between two chains of about 3 Å was estimated by determining energy minima using molecular dynamics. For this geometry, the *I*–*V* characteristics were computed and compared with results for one PANI chain. From the linear part of this characteristic, the resistance and, thus, the resistance change compared to the one-chain case was computed. Next, the same geometry with one gas molecule was computed. The resistance change results are displayed in Figure 7.

The hopping resistance change is rather large in comparison to the resistance change due to the presence of ammonia gas molecules. But one can see the effect of the ammonia gas molecule as well. The resistance increase is higher for the ammonia position near the doped polyaniline nitrogen and lower at the undoped position. Due to higher resistance of the hopping process, the current is flowing mainly through the direct connection of the sensor electrodes through unperturbed PANI chains. Therefore, the hopping influence can be ignored in the comparison with the experimental data.

### Limitations and validity of the model

The extended Hückel method is a semi-empirical method and cannot display reality completely. To compare this model with experimental results, consideration of the computational limits is crucial. The molecule models consists only of one or two PANI molecule chains with a transmission area of 166 Å. These chains are interacting with only several gas molecules. These conditions affect the *I*–*V* characteristics. They affect its slope and the resistance effect of ammonia/nitrogen dioxide gas on polyaniline. The abovementioned current saturation in the *I*–*V* characteristics arises from the one or two relatively short chains that were used for computation. That is, only a small, limited quantity of free charge carriers is available. Also, as seen in the *I*–*V* characteristic obtained through the extended Hückel method, in the saturated part, ballistic charge carrier transport of one particle takes place. Thus, for the relative resistance change data, only the initial part of the *I*–*V* diagram was taken into account. Also, the numerical experiment models only a small region and, therefore, the results are influenced by the limitations of the modeled environment.

The gas concentration in ppm for the numerical experiment was calculated approximately as the next step. In this case, one gas molecule in the active area of the device was computed using dimensions of the polyaniline polymer to correspond to about 3 ppm concentration.

Finally, in this numerical model many effects are not taken into account, among others, the fact that the bulk material contains a large number of polymer chains and that there are, for example, kinetic effects and inter-carrier influences. Therefore, polyaniline bulk material would have a slightly different resistance values than the computed model.

### Comparison with experimental data

Numerical models for a one-chain polyaniline sensor were compared with experimental data. Chemiresistive gas sensors for ammonia and nitrogen dioxide containing a flexible PANI thin film sensing area deposited on interleaved electrodes were produced by Posta et al. [13] and by Kroutil and co-workers [7]. In the experiments of Posta and Kroutil, the gas sensors for ammonia and nitrogen dioxide were exposed for 20 min to synthetic air with defined concentrations of NH_3_ and NO_2_. Subsequently, they were exposed to clear synthetic air without added gases for the next 20 min.

**Figure 8:**
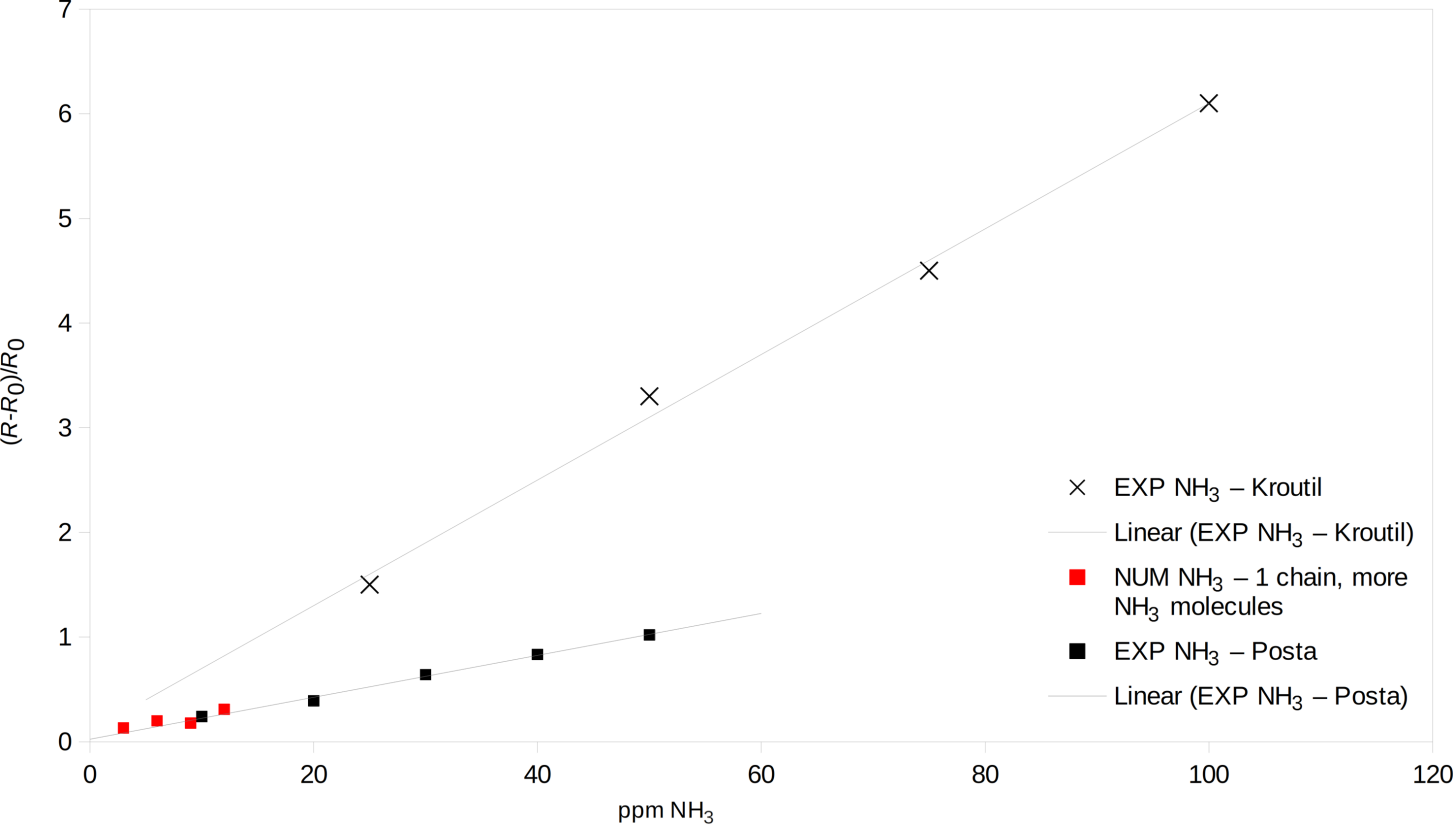
Relative resistance change in the presence of ammonia for different concentrations; red squares: computation, black crosses: experimental data form [7], black squares: experimental data from [13].

**Figure 9:**
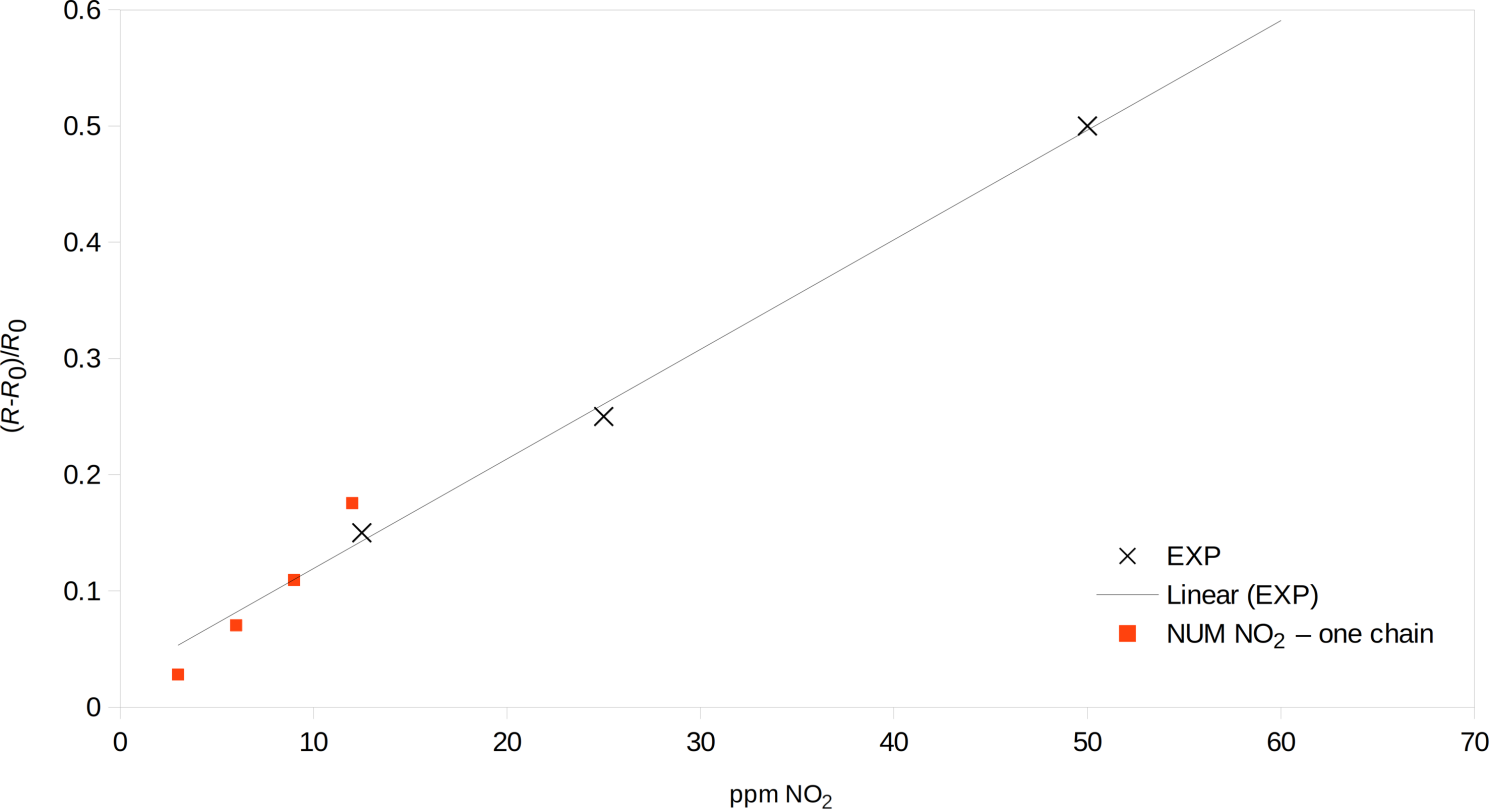
Relative resistance change in the presence of nitrogen dioxide for different concentrations; red squares: computation, black crosses: experimental data from [7].

The experimental results were fitted for saturation values and the results were compared with the computed resistance values (Figure 8 and Figure 9). The modeled resistance values of polyaniline fit the experimental results rather well.

Comparing both experiments of Kroutil et al. and Posta et al. (Figure 8), unlike linear dependencies are found. The mismatch of the absolute values comes from the difference of the experimental setups. Different samples with different polyaniline resistance values led to unequal resistance differences.

The numerical values for NO_2_ in Figure 9 have slightly stronger slopes than the linear trend of the experimental data. This effect arises from the limitation of the numerical model as explained above. In the experiment, the PANI sensor was 100 μm wide and overlaying molecules contributed to the conductivity, sharing their charge carriers and allowing for carrier hopping, while the numerical experiment consisted of only one, 166 Å long molecule with non-interacting charge carriers. Considering these limitations, the agreement between the experiment and the numerical model is good.

## Conclusion

Several models of polyaniline emeraldine salt interacting with ammonia and nitrogen dioxide gas were established, and their *I*–*V* characteristics were computed through numerical modeling (Synopsys QuantumATK [14]) to obtain the resistance of these one-chain and two-chain systems. From these *I*–*V* characteristics of emeraldine salt molecules in several configurations, including hopping, the relative resistance, (*R* − *R*_0_)/*R*_0_, was computed and compared with experimental data from [7,13]. These one-chain and two-chain systems provide data about the importance of the gas molecule position relative to the conducting chain, the contribution of hopping effects for the conductivity, and to which extent the resistance depends on the geometry of the polymer molecule. The relative change of conductivity of the emeraldine salt was found both for pure PANI and for PANI with adsorbed ammonia and nitrogen dioxide molecules corresponding to gas concentrations of 3, 6, 9, and 12 ppm. The two-chain model was computed with only one gas molecule and, thus, the concentration was not varied.

First, the resistance of the system with adsorbed NH_3_ and/or NO_2_ molecules increases. The discontinuity principally affects the resistance increase. Much smaller, but still significant, is the relative resistance increase for the system with ammonia compared to the system without ammonia, where the largest effect was found for ammonia molecules placed near the doped nitrogen atoms of the emeraldine salt. A smaller effect on the resistance has the presence of nitrogen dioxide molecules. Like for ammonia, the resistance change depends strongly on the position of the nitrogen dioxide molecules. A gas molecule near the doped nitrogen makes the polyaniline chain more resistant than one near at the undoped position, and the resistance change of several nitrogen dioxide molecules at different positions (near the doped/undoped polyaniline nitrogen) is rather the sum of the resistance changes of both positions.

The relative resistance increase for a one-chain system with several gas molecules calculated in vacuum was compared to experiments both for NH_3_ and NO_2_ in synthetic air and the values were found to be comparable. The calculated resistance values seem to fit the experimentally obtained values well, and the presence of other molecules in synthetic air do not significantly affect the response. Polyaniline emeraldine salt seems to be usable for detecing NH_3_ and NO_2_, although the resistance change caused by NO_2_ gas is much lower than that caused by ammonia.
